# Wearable sensors in paediatric neurology

**DOI:** 10.1111/dmcn.16239

**Published:** 2025-01-31

**Authors:** Camila González Barral, Laurent Servais

**Affiliations:** ^1^ Sysnav Vernon France; ^2^ Neuromuscular Reference Center, Department of Pediatrics University Hospital Liège Belgium; ^3^ Faculty of Medicine, Department of clinical sciences University of Liège Liège Belgium; ^4^ MDUK Oxford Neuromuscular Centre University of Oxford Oxford UK; ^5^ NIHR Oxford Biomedical Research Centre University of Oxford Oxford UK

## Abstract

Wearable sensors have the potential to transform diagnosis, monitoring, and management of children who have neurological conditions. Traditional methods for assessing neurological disorders rely on clinical scales and subjective measures. The snapshot of the disease progression at a particular time point, lack of cooperation by the children during assessments, and susceptibility to bias limit the utility of these measures. Wearable sensors, which capture data continuously in natural settings, offer a non‐invasive and objective alternative to traditional methods. This review examines the role of wearable sensors in various paediatric neurological conditions, including cerebral palsy, epilepsy, autism spectrum disorder, attention‐deficit/hyperactivity disorder, as well as Rett syndrome, Down syndrome, Angelman syndrome, Prader–Willi syndrome, neuromuscular disorders such as Duchenne muscular dystrophy and spinal muscular atrophy, ataxia, Gaucher disease, headaches, and sleep disorders. The review highlights their application in tracking motor function, seizure activity, and daily movement patterns to gain insights into disease progression and therapeutic response. Although challenges related to population size, compliance, ethics, and regulatory approval remain, wearable technology promises to improve clinical trials and outcomes for patients in paediatric neurology.

## Graphical abstract

Types of signals monitored in children's natural environments using wearable sensors, and their associated applications in various paediatric neurological conditions.

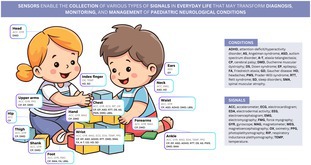

Plain language summary: https://onlinelibrary.wiley.com/doi/10.1111/dmcn.16267

**Abbreviations**
ASD: autism spectrum disorder
DMD: Duchenne muscular dystrophy
EDA: electrodermal activity
HRV: heart rate variability
PPG: photoplethysmography
RTT: Rett syndrome
SMA: spinal muscular atrophy
SV95C: stride velocity 95th centile

**What this paper adds**

- Recent advancements in wearable sensors highlight their potential to transform the diagnosis, monitoring, and management of paediatric neurological conditions.
- More research is needed to validate the effectiveness and clinical impact of wearable sensors.
- Regulatory approval or even formal validation of digital outcomes is extremely rare.

Over the past two decades, there has been growing interest in developing treatments for neurological conditions because of the significant impact of these diseases on global health. A recent study showed that 3.4 billion individuals, representing approximately 43.1% of the world's population, are affected by conditions that affect the nervous system.^1^ Given the short timeframes of clinical trials, several drugs have been approved on the basis of biomarkers, such as micro‐dystrophin level in Duchenne muscular dystrophy (DMD).^2^

Traditionally, neurological disease progression and treatment efficacy have been assessed using methods such as clinical scales, questionnaires, and patients' diaries. However, these approaches present reliability and consistency issues due to their subjective nature. Additionally, these methods typically capture a single point in time, posing significant challenges in evaluating rapidly progressing diseases, such as Krabbe disease, fluctuating conditions such as myasthenia gravis, and diseases influenced by patients' fatigue or motivation, such as spinal muscular atrophy (SMA) and DMD. Furthermore, the requirement for repeated visits to clinical sites for assessments can discourage participation in clinical trials.

To address these limitations, there is increasing interest in developing objective and less burdensome evaluation methods that could be used in clinical trials, in diagnosis, and in monitoring of therapy efficacy. Wearable sensors offer a promising solution for non‐invasive monitoring of neurological disorders. These devices, placed on shoes, eyeglasses, clothing, or watches, can collect a range of biological signals (e.g. heart rate, blood pressure) and track movement, while also assessing cognition, speech, and pain.^3^

One key advantage of wearable sensors is their capacity to collect data in real‐world settings, offering a more comprehensive understanding of disease progression. Sensors can be used in active assessment, when the patient is asked to perform the same task on a regular basis, or as a passive assessment, when daily activity is recorded. The COVID‐19 pandemic accelerated the adoption of wearable technologies, particularly for monitoring cardiovascular, respiratory, and activity data.^4^, ^5^ This review focuses on the role of wearable technology in paediatric neurology.

## METHOD

The PubMed electronic database was used to identify relevant published articles, using keywords such as ‘wearable sensors’, ‘child’, and ‘neurological disorder’. After identifying key neurological conditions studied using wearable sensors (such as cerebral palsy [CP] and epilepsy), a new search was conducted for each condition. A total of 209 result titles and abstracts were screened for inclusion on the basis of the mention of the use of wearable sensors in children living with a neurological disorder. Reviews and studies that did not include children were not considered. Full‐text articles of shortlisted studies were then assessed for adherence to the review question. This process resulted in 84 articles. We note that this was not a systematic review as the search was conducted by a single assessor and using a single database.

## RESULTS

**FIGURE 1:**
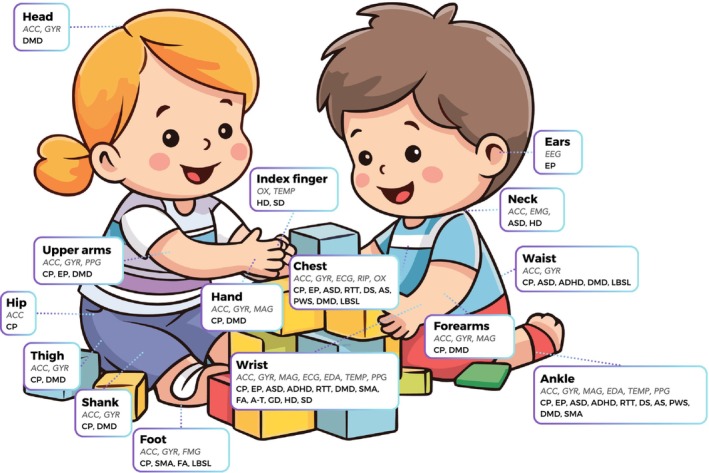
Types of signals monitored in children's daily activities and their associated applications in various paediatric neurological conditions. Abbreviations: ACC, accelerometer; ADHD, attention‐deficit/hyperactivity disorder; AS, Angelman syndrome; ASD, autism spectrum disorder; A‐T, ataxia–telangiectasia; CP, cerebral palsy; DMD, Duchenne muscular dystrophy; DS, Down syndrome; ECG, electrocardiogram; EDA, electrodermal activity; EEG, electroencephalogram; EMG, electromyography; EP, epilepsy; FA, Friedreich ataxia; FMG, force myography; GD, Gaucher disease: HD, headaches; GYR, gyroscope; MAG, magnetometer; MEG, magnetoencephalography; OX, oximetry; PPG, photoplethysmography; PWS, Prader–Willi syndrome; RIP, respiratory inductance plethysmography; RTT, Rett syndrome; SD, sleep disorders; SMA, spinal muscular atrophy; TEMP, temperature.

Tables 1, 2, 3, 4, 5, 6, 7, 8 summarizes how wearable sensors have been applied in paediatric neurology research. Figure 1 illustrates the use of wearable devices in children's daily lives and the wide range of signals that can be monitored for each reviewed pathology.

**TABLE 1:** Summary of uses of wearable sensors in cerebral palsy research.

Reference	Sensor	Population (age range)	Position	Data acquisition	Setting	Findings
Franchi de’ Cavalieri et al.^6^	Axivity AX3 (Axivity)	Seventeen with CP	Wrists and chest	ACC	At home for up to 8 weeks during daily rehabilitation training	Activity levels, derived from acceleration data, can predict clinical scales.
von Gunten et al.^7^	Axivity AX3 (Axivity)	Eight with CP (up to 1 year)	Upper arms	ACC	At home twice a week for 6 weeks during normal daily activities and upper limb assessments	Preliminary findings suggest wearable sensors can monitor upper limb function during action observation training for high‐risk unilateral CP infants. Action observation training combined with sensor measurements offers a feasible training tool to supplement standard care.
den Hartog et al.^8^	DOT (Xsens Technologies)	Twelve with CP (10–21 years)	Wrist, ankle	ACC, GYR, MAG	At home or in the hospital for 7 weeks during activities such as walking or gaming	Assessing dystonia in dyskinetic CP outside clinical settings is feasible using home measurements and personalized machine learning models. The sensors provide clinically useful information on dystonia progression over longer periods.
Strohrmann et al.^9^	ETH Orientation Sensor (ETHOS)	Two with CP (9–11 years)	Upper arms, wrists, chest, hip, thighs, foot	ACC, GYR	In a clinical setting once a week for 4 weeks during standardized motor tasks	Wearable sensors can reliably evaluate task capacity and estimate movement capacity across various tasks. Three sensors, one on each wrist and one on the hip, provided sufficient information for assessing daily life activities and motor performance in patients with CP.
Hegde et al.^10^	FSR sensors (Interlink)	Ten with CP and 11 typically developing individuals (4–9 years)	Foot	FMG, ACC	In a clinical setting during assessment of sitting, standing, and walking	A footwear‐based wearable system, integrated with a machine learning algorithm, accurately monitored the activity and gait of individuals with CP.
Ahmadi et al.^11^	GT3X Actigraph, (Actigraph Corporation)	Twenty‐two with CP (6–20 years)	Hip, wrist	ACC	In a clinical setting during assessment of daily activities such as walking and sitting	Machine learning methods achieved acceptable classification accuracy in detecting various activities commonly performed by ambulatory individuals with CP.
Ahmadi et al.^12^	GT3X Actigraph, (Actigraph Corporation)	Thirty‐eight with CP (6–18 years)	Wrist, hip, and ankle	ACC	In a clinical setting during assessment of walking and sitting	In laboratory conditions, personalized activity classification models (on individual data) outperform group models in recognizing physical activity in individuals with CP, especially those with severe impairments. Under simulated free‐living conditions, personalized models show higher accuracy than group models, but performance of all models declined significantly.
Beani et al.^13^	GT3X Actigraph, (Actigraph Corporation)	Fifty with CP and 50 typically developing individuals (3–25 years)	Wrists	ACC	In a clinical setting during Assisting Hand Assessment	Actigraphy can quantitatively describe differences in upper limb activity in a standardized setting.
Yazıcı et al.^14^	G‐Walk (BTS Bioengineering Company)	Fifty‐four with CP (5–15 years)	Waist	ACC	In a clinical setting during walking tasks at baseline and 5 days later	The intraclass correlation coefficient test (0.799–0.977 for all gait parameters) indicated that gait analysis conducted with the G‐Walk system is a reliable way to analyse gait in children with CP in a clinical context.
Burgess et al.^15^	GT3X (Actigraph Corporation)	Forty‐four with CP (6–12 years)	Wrists	ACC	In a clinical setting during Both Hands Assessment	Significant and positive correlations were found between the Both Hands Assessment and mean activity counts.
Wolff et al.^16^	MTw Awinda (XSens)	Fourteen with CP (3–28 years)	Wrists	ACC, GYR, MAG	In a clinical setting during assessment of three self‐selected gait speeds: walking, fast walking, and running	Inertial sensors can measure variations in arm movements and detect asymmetry changes across different gait speeds in individuals with CP.
Vanmechelen et al.^17^	MTw Awinda (XSens)	Fifteen with dyskinetic CP and eight typically developing (means 16 and 17 years respectively)	Arm, forearm, and hand	ACC, GYR	In a clinical setting during monitoring of reach‐forward, reach‐and‐grasp‐vertical, and reach‐sideways tasks	Sensor‐derived parameters reliably capture pathological movements in individuals with dyskinetic CP. Between‐group differences demonstrate the ability to discriminate between pathological and non‐pathological movement patterns, offering opportunities for further exploration of these patterns in individuals with dyskinetic CP.
Choi et al.^18^	RAPAEL Smart Kids (Neofect Co.)	Seventy‐eight with CP (3–16 years)	Dorsum of the hand and forearm	ACC, GYR, MAG	In a clinical setting during 20 sessions of virtual reality intervention followed by conventional occupational therapy, over 4 weeks	A multi‐centre, single‐blind, randomized controlled trial showed both groups improved, but the virtual reality group led to greater gains in dexterity, daily activities, and forearm supination than typically developing comparison individuals. Severe motor impairment children showed significant improvements.
Brégou Bourgeois et al.^19^	Physilog1 (GaitUp)	Fourteen with CP and 15 typically developing (6–15 years)	Foot	ACC, GYR	In a clinical setting for a 200‐metre walk at a self‐determined normal pace	Foot‐worn inertial sensors enable analysis of temporal and spatial gait parameters with good accuracy and precision, revealing expected gait variations in CP including reduced speed, stride length, longer stance phases, and decreased foot strike and lift‐off pitch angles.
Choi et al.^20^	Inertial sensor	Twenty‐eight with CP (mean age 6.75 years, SD 3.24) and five typically developing (mean 26.0, SD 2.0)	Shank, thigh, foot	ACC, GYR	In a clinical setting during Modified Tardieu Scale assessment	Wearable sensors significantly enhance the accuracy and reliability of the Modified Tardieu Scale for lower limb assessment in individuals with CP.
Baram et al.^21^	Inertial sensor	Ten CP and typically developing individuals (5–26 years)	Waist	ACC, GYR	In a clinical setting during walking tests	Training aided by wearable sensor‐driven visual and auditory feedback cues enhances gait parameters in patients with CP‐related gait disorders. No improvement was observed in age‐matched typically developing individuals.
Brégou Carcreff et al.^22^	Physilog4 (GaitUp)	Fifteen CP and 11 typically developing individuals (8–20 years)	Shanks, thighs, feet	ACC, GYR	In a clinical setting during a 10‐metre walk test	Using sensors on both feet produced greater accuracy for typical gait patterns (typically developing individuals), whereas shank and thigh sensors were more effective for moderate to severely impaired gait patterns (CP with Gross Motor Function Classification System levels II and III). Thus, inertial sensors show promise for objectively evaluating daily life gait.

Abbreviations: ACC, accelerometer; CP, cerebral palsy; FMG, force myography; GYR, gyroscope; MAG, magnetometer; SD, standard deviation.

**TABLE 2:** Summary of uses of wearable sensors in epilepsy and seizure research.

Reference	Sensor	Population (age range)	Position	Data acquisition	Setting	Findings
Vieluf et al.^23^	E4 (Empatica)	Sixty‐six with epilepsy (up to 27 years)	Wrist or ankle	EDA, TEMP, PPG	In a clinical setting during long‐term video‐EEG monitoring	Changes in states related to epilepsy were detected using peripheral wearable devices.
Yamakawa et al.^24^	RRI telemeter prototype	Seven with epilepsy (9–54 years)	Chest	ECG	In a clinical setting during long‐term video‐EEG monitoring	EEG monitoring predicts seizures in real‐time, suggesting possible applications in real‐world settings.
El Atrache et al.^25^	E4 (Empatica)	Eleven with epilepsy (7–15 years)	Either both wrists or both ankles	PPG	In a clinical setting during long‐term video‐EEG monitoring	PPG alterations are observable before, during, and after focal impaired awareness seizures.
Arends et al.^26^	Nightwatch (LivAssured)	Thirty‐four with epilepsy (15–67 years)	Upper arm	PPG, ACC	At home for 2–3 months	Combining heart rate and movement monitoring proved to be effective in reliably detecting a diverse range of nocturnal seizures.
Hegarty‐Craver et al.^27^	Bittium Faros 180 (Zephyr Biopatch)	Sixty‐two with epilepsy (2–17 years)	Chest	ECG, ACC	In a clinical setting during long‐term video‐EEG monitoring	Cardiac measures could identify seizures with bilateral motor features with high sensitivity.
Proost et al.^28^	Sensor Dot (Byteflies)	Forty‐nine with epilepsy (4–18 years)	Behind the ears, upper arm, chest	EEG, ACC, GYR, EMG ECG	In a clinical setting during long‐term video‐EEG monitoring	The sensitivity of seizure diaries decreased at night but increased at night for the wearable device. Using a multimodal wearable device with multiple adhesive sites is feasible for individuals with epilepsy and intellectual disabilities.
Meisel et al.^29^	E4 (Empatica)	Sixty‐nine with epilepsy (up to 27 years)	Wrist or ankle	EDA, TEMP, PPG, ACC	In a clinical setting during long‐term video‐EEG monitoring	Machine learning achieved better‐than‐chance performance in predicting seizures in approximately half of the patients.
Tang et al.^30^	E4 (Empatica)	Ninety‐four with epilepsy (median years = 9.9, range 27.2, interquartile range 9.2)	Either the wrist or ankle	EDA, TEMP, PPG, ACC	In a clinical setting during long‐term video‐EEG monitoring	Machine learning algorithms utilising data from wearable sensors detected various types of epileptic seizure.
Yu et al.^31^	E4 (Empatica)	One hundred and sixty‐six with epilepsy (median years = 10.0, range 29.6, interquartile range 8.3)	Either the wrist or ankle	EDA, PPG, ACC	In a clinical setting during long‐term video‐EEG monitoring	Deep learning algorithms, trained on data from wearable sensors, identified 28 distinct types of seizure.
Bisi et al.^32^	Opal (APDM)	Twenty with CP and 112 typically developing (6–33 years)	Lower back and ankles	ACC, GYR	In a clinical setting while walking back and forth along a straight path	Gait parameters reflected the unsteady, ataxic gait of individuals with Dravet syndrome.
Mohammadpour Touserkani et al.^33^	E4 (Empatica)	Thirteen with epilepsy (9–27 years)	Wrist or ankle	PPG	In a clinical setting during long‐term video‐EEG monitoring	PPG is a promising biomarker for seizure detection, showing increased frequency during both pre‐ and post‐seizure periods compared with seizure‐free intervals.
Swinnen et al.^34^	Sensor Dot (Byteflies)	Twelve with CP (8–50 years)	Behind the ears	EEG	In a clinical setting during long‐term video‐EEG monitoring	The study found that the use of Sensor Dot could significantly reduce the review time of video‐EEG recordings from 1 to 2 hours to around 5–10 minutes.

Abbreviations: ACC, accelerometer; CP, cerebral palsy; ECG, electrocardiogram; EDA, electrodermal activity; EEG, electroencephalogram; EMG, electromyography; GYR, gyroscope; PPG, photoplethysmography; TEMP, temperature.

**TABLE 3:** Summary of uses of wearable sensors in ASD research.

Reference	Sensor	Population (age range)	Position	Data acquisition	Setting	Findings
Konrad et al.^35^	GT3X (Actigraph Corporation)	Twenty‐two ASD and 26 typically developing individuals (3–10 years)	Wrists	ACC	At home for 12 hours	Sensor variables from ASD and typically developing children revealed age‐related changes in movement with intensity and complexity linked to motor coordination. This suggests that wearable sensors could offer valuable tools for measuring motor characteristics in individuals with ASD.
Wilson et al.^36^	Opal (APDM)	Five at high risk for ASD (up to 1 year)	Ankles	ACC	At home for 1 full day at 3, 6, 9, and 12 months of age.	Motion complexity could track early infant motor development and identify high‐risk infants likely to develop ASD.
Wilson et al.^37^	E4 (Empatica)	Nineteen with ASD, 17 with ADHD, and 82 typically developing (up to 36 months)	Ankles	ACC	In a clinical setting during assessments at 12, 18, 24, and 36 months of age	Movement curvature predicted ASD at 18, 24, and 36 months, with less variable movements linked to later ASD diagnosis.
Gilchrist et al.^38^	Axivity AX3 (Axivity) and Zephyr Biopatch	Twenty with intellectual and developmental disabilities (12–31 years)	Wrists and neck for AX3 and chest for Biopatch	ACC	In a clinical setting during a set of activities involving hand and body motions	Automated detection of repetitive behaviours is feasible, promising help in identifying treatments and monitoring changes over time and in response to clinical trials.
Min^39^	Custom‐designed accelerometer	Four with ASD	Wrist, back below the neck	ACC	In therapy sessions and at home during hand flapping, punching, drumming, and rocking	Wearable sensors may offer objective insights into the triggers of self‐stimulatory patterns in individuals with autism.
Cantin‐Garside et al.^40^	GT9X Link (Actigraph Corporation)	Eleven with ASD (5–14 years)	Wrists, waist, pockets, and/or ankles	ACC	In a clinical setting during free play	Wearable sensors demonstrate accuracy, specificity, and efficiency in detecting various self‐injurious behaviours, indicating the feasibility of an effective monitoring system for these behaviours, a critical concern in ASD.
Siddiqui et al.^41^	Flex sensor	Ten with ASD	Wrist	ACC, GYR	In a clinical setting while patients performed typical ASD gesture	The use of wearable sensors allows for the monitoring and classification of gestures in children with ASD with an accuracy of about 91%.
Puli et al.^42^	Shimmer 2r	Fifteen with ASD (mean age 14 years, SD 2)	Chest	ECG, ACC	In a clinical setting during anxiety‐inducing activities	The real‐time algorithm minimized false detections due to motion and accurately identified arousal states during movement.
Zheng et al.^43^	E4 (Empatica) and WINGS	Seven with ASD (4–15 years)	Wrist and upper body respectively	EDA, PPG, ACC and ACC, GYR, MAG respectively	In a clinical setting during activities designed to induce problem behaviours	The PreMAC framework showed high prediction accuracy for detecting precursors of problem behaviours, indicating potential to alert caregivers. Body motion resulted the most predictive sensing modality.
Deng et al.^44^	Apple Watch (Apple)	Thirty with ASD and 30 typically developing preschool infants	Wrist	ACC, ECG	In classroom setting during attention tasks	Using sensory data and machine learning, individuals' stress and attention levels were predicted aiding in sensory management.
Ali et al.^45^	Samsung Galaxy Watch 3 (Samsung)	Nine with ASD (8–11 years)	Wrist	ECG	In a simulation of an in‐the‐wild setting, while interacting with an avatar	The proposed approach achieves similar performance levels as cutting‐edge heart‐rate‐based emotion recognition methods.

Abbreviations: ACC, accelerometer; ADHD, attention‐deficit/hyperactivity disorder; ASD, autism spectrum disorder; ECG, electrocardiogram; EDA, electrodermal activity; GYR, gyroscope; MAG, magnetometer; PPG, photoplethysmography.

**TABLE 4:** Summary of uses of wearable sensors in ADHD research.

Reference	Sensor	Population (age range)	Position	Data acquisition	Setting	Findings
Lindiem et al.^46^	Apple Watch (Apple)	Fifteen ADHD and 15 typically developing individuals (6–11 years)	Wrist	ACC	At home for 2 days	Sensors offer practical, cost‐effective solutions with high usability scores for objectively measuring hyperactivity in children, achieving a diagnostic accuracy of 0.89 (sensitivity = 0.93; specificity = 0.86).
Faedda et al.^47^	Mini‐motionlogger or motionlogger watch (Ambulatory Monitoring)	Forty‐eight with bipolar disorder, 44 ADHD, 21 ADHD plus comorbid depressive disorder, and 42 typically developing individuals (5–18 years)	Waist	ACC	At home and at school for 3–5 days	Wearable sensor technology can potentially provide bio‐behavioural markers to distinguish children with bipolar disorder from those with ADHD and healthy controls.
Muñoz‐Organero et al.^48^	Runscribe inertial sensors (Scribe Labs)	Eighteen ADHD and 18 typically developing individuals (6–16 years)	Wrists and ankles	ACC	At home and at school for 24 consecutive hours	Non‐medicated ADHD participants exhibited significantly different movement patterns compared with typically developing peers during medium‐intensity activities. In contrast, medicated participants with ADHD showed statistically significant differences in low intensity movements.
Park et al.^49^	GT3X (Actigraph Corporation)	Thirty‐nine with ADHD (7–16 years)	Waist	ACC	At home and at school for 7 days at baseline, 6 months, 12 months	Faster movements, measured using wearable sensors, combined with age, height, and Child Behavioral Checklist total score, could help identify physically aggressive incidents using machine learning.

Abbreviations: ACC, accelerometer; ADHD, attention‐deficit/hyperactivity disorder.

**TABLE 5:** Summary of uses of wearable sensors in RTT research.

Reference	Sensor	Population (age range)	Position	Data acquisition	Setting	Findings
Leoncini et al.^50^	YouCare Wearable Medical Device (Accyourate Group SpA)	Ten with RTT (4–35 years)	Chest (smart T‐shirt)	ECG	At home for 24 hours	Data accuracy exceeded 70%, with no major adverse events. HRV metrics correlated with age, clinical severity, and *MECP2* mutations. Percentage of maximum heart rate and heart rate to HRV low‐frequency power ratio were identified as fatigue markers.
Gualniera et al.^51^	E4 (Empatica)	Ten with RTT (6–20 years)	Wrist	ACC, PPG, TEMP	In a clinical setting for 30–90 minutes in eight patients, and 10–12 hours in two patients at baseline and follow‐up visits, after periods spanning 4–28 months	EDA may provide objective and reliable measure of autonomic dysregulation.
Migovich et al.^52^	E4 (Empatica)	Seven (4–16 years)	Wrist	ACC, PPG, TEMP	At home for 2 days	Machine learning models achieved 85.1% accuracy in distinguishing awake, non‐rapid eye movement sleep, and rapid eye movement sleep stages. Incorporating physiological data notably improved predictions, especially for rapid eye movement sleep, which was less accurately detected using only accelerometer data.
Singh et al.^53^	E4 (Empatica)	Forty‐five (3–41 years)	Wrist	PPG	During the day and night	Mean heart rate decreased with age in individuals with RTT, and was lower at nigh across the entire age range in patients with RTT.
Carroll et al.^54^	LifeShirt (VivoMetrics)	Forty‐seven RTT and 47 typically developing individuals (2–7 years)	Chest	ECG, RIP, oximetry	At home for one night and two daytime recording 2‐hour sessions	HRV measures are consistently lower in patients with RTT, who may exhibit elevated sympathetic tone during the day or reduced parasympathetic tone at night, compared with typically developing individuals. HRV metrics detect subtle differences across *MECP2* variant subclasses.
Iakovidou et al.^55^	E4 (Empatica)	Ten with RTT and 10 with ASD (9–19 years)	Wrists or ankles	ACC, PPG, TEMP	In a clinical setting	Patients with ASD exhibited increased dysfunction in EDA, while patients with Rett syndrome showed higher levels of HRV and motor activity dysregulation. The results indicate a 95% accuracy in distinguishing between ASD and Rett syndrome.

Abbreviations: ACC, accelerometer; ASD, autism spectrum disorder; ECG, electrocardiogram; EDA, electrodermal activity; HRV, heart rate variability; PPG, photoplethysmography; RIP, respiratory inductance plethysmography; RTT, Rett syndrome; TEMP, temperature.

**TABLE 6:** Summary of uses of wearable sensors in Down syndrome, Angelman syndrome, and PWS research.

Reference	Sensor	Population (age range)	Position	Data acquisition	Setting	Findings
Belluscio et al.^56^	Opal (APDM)	Fifteen with Down syndrome, 11 with PWS, and 12 typically developing individuals (2–11 years)	Ankles, chest, and pelvis	ACC, GYR	In a clinical setting during standardized gait assessments	Compared with typically developing individuals, those with Down syndrome and PWS exhibited altered walking strategies. Since they often receive similar treatments, this study's results may assist clinicians in creating a more comprehensive and patient‐specific clinical picture.
Khasgiwale et al.^57^	Opal (APDM)	Nine with Down syndrome and nine typically developing (3–5 months)	Ankles	ACC	At home five times a week for 8 weeks	Infants with Down syndrome showed increased leg movement rates after in‐home kick‐toy intervention, approaching rates seen in typically developing infants.
Kraan et al.^58^	Physilog5 (GaitUp)	Five with Angelman syndrome and nine with PWS (6–16 years)	Ankles	ACC, GYR	In clinical setting during assessments and ‘real‐world’ long walk	Metrics such as mean stance percentage, mean stride length, and stance percentage coefficient of variation from ‘real‐world’ assessments closely aligned with laboratory results, demonstrating the feasibility of ‘real‐world’ gait analysis in children with PWS and Angelman syndrome.
Duis et al.^59^	ActiMyo (Sysnav)	Five with Angelman syndrome and 45 typically developing (4–11 years)	Ankles	ACC, GYR, MAG	At home for 14 days	Children with Angelman syndrome demonstrated good tolerability for wearing sensors for several hours daily. Compared with typically developing age‐matched children, those with Angelman syndrome walked less, with shorter stride lengths and higher median stride duration. They also showed decreased SV95C and, on average, a lower coefficient of variability in stride velocity.

Abbreviations: ACC, accelerometer; GYR, gyroscope; MAG, magnetometer; PWS, Prader–Willi syndrome; SV95C, stride velocity 95th centile.

**TABLE 7:** Summary of uses of wearable sensors in DMD research.

Reference	Sensor	Population (age range)	Position	Data acquisition	Setting	Findings
McErlane et al.^60^	Custom‐designed accelerometer (Aparito)	Eight with DMD (9–15 years)	Wrist	ACC	At home for 12 weeks	Wearable data capture provides a continuous view of physical activity patterns, complementing outcomes obtained during clinic visits, allowing researchers to gain a deeper understanding of how DMD affects daily life.
Ramli et al.^61^	iPhone 11 MEMS accelerometer (STMicroelectronics)	Fifteen with DMD and 15 typically developing (aged 3–16 years)	Waist	ACC	In a clinical setting during standardized gait assessments and free walk	A machine learning approach effectively detects strides and stride lengths by analysing individual gait patterns during clinical assessments and when participants walk at their own pace.
Ramli et al.^62^	iPhone 11 MEMS accelerometer (STMicroelectronics)	Fifteen with DMD and 15 typically developing (3–16 years)	Waist	ACC	In a clinical setting during standardized gait assessments and free walk	Machine learning and deep learning models applied to gait assessments distinguished between children with DMD and typically developing individuals based on their gait patterns, achieving accuracy levels of up to 100%.
Lott et al.^63^	GT3X Actigraph, (Actigraph Corporation)	Seventy with DMD and 10 typically developing (5–12 years)	Waist	ACC	At home for 7 days	Male children with DMD took 63% fewer daily steps than typically developing male children. Among males with DMD, there was a trend of decreasing step activity with age. A correlation existed between daily step count with functional capacity and with overall strength in males with DMD. Those remaining ambulatory after 2 years had baseline step activity nearly twice as high as those who stopped walking.
Ganea et al.^64^	Autonomous Sensing Unit Recorder (LMAM/EPFL) and Physilog (BioAGM)	Twenty‐five with DMD and 20 typically developing (5–12 years)	Shanks and chest respectively	ACC	In a clinical setting for a 200‐metre walk at a self‐determined normal pace	Patients with DMD had lower stride length, velocity, and higher double support time than typically developing individuals. Correlations exist between clinical scores and gait parameters. Classification using select parameters achieved 80% accuracy distinguishing patients with mild and moderate DMD.
Jeannet et al.^65^	Non‐commercialized miniaturized data logger (Autonomous Sensing Unit Recorder)	Five with DMD (4–6 years)	Chest	ACC, GYR	At home for 2 days at baseline and 2 days a month later	A chest‐worn device can accurately track a wide range of daily activity details in patients with DMD.
Kimura et al.^66^	Motionlogger Watch (Ambulatory Monitoring)	Twenty with DMD (4–19 years)	Wrist	ACC	At home for 7 days	Muscle strength can be estimated using activity level.
Fowler et al.^67^	StepWatch Activity Monitor (Modus Health)	Forty‐two with DMD (4–16 years)	Ankle	ACC	At home for 3 to 5 weekdays and two weekend days every 6 months over a 5‐year period	The decrease in average strides per day with age mirrored the trend seen in other functional metrics for DMD.
Arteaga et al.^68^	GT3X Actigraph, (Actigraph Corporation)	Forty‐nine with DMD and 15 typically developing (8–24 years)	Wrist, ankle	ACC	At home for 7 days	Participants with DMD were mostly inactive. Those who could walk were more active than those who could not. Activity levels in all individuals with DMD were lower than typically developing individuals and were lower in non‐ambulatory than ambulatory patients. Age and ability to walk affected activity levels more than steroid use.
Killian et al.^69^	GT3X Actigraph, (Actigraph Corporation)	Forty‐eight with DMD (13.0 ± 4.3 years)	Wrist, ankle	ACC	At home for 7 days at baseline, and at 1 year and 2 years	Individuals with DMD showed progressive decrease in physical activity. Total daily activity correlated with skeletal muscle strength.
Siegel et al.^70^	Actiwatch 2 (Philips Respironics)	Fifty‐four with DMD (5–17 years)	Wrist	ACC	At home for 10 days	Eleven participants (20%) experienced pathological sleep, which was linked to reduced quality of life but not with ambulatory status. Among ambulatory participants, disruption in rest–activity rhythms was connected to perceived sleep difficulties. Daytime activity was linked with performance in the 6MWT.
Van der Geest et al.^71^	Moximetry Accelerometry (Maastricht Instruments)	Sixteen with DMD (7–17 years)	Upper arm, lower arm, wheelchair	ACC	At home for 1–3 days	The level of upper limb activity and transfer frequency correlates with arm function metrics (Brooke and Performance of Upper Limb scales). Accelerometer data on upper limb activity during daily life mirror self‐reported activity, validating accelerometry for assessing daily activity levels.
Le Moing et al.^72^	ActiMyo (Sysnav)	7 patients with DMD (10–28 years)	Wrists	ACC, GYR, MAG	In a clinical setting during standardized assessments at baseline and 7 days later	A prototype of the ActiMyo reliably measured hand and arm movement, with good repeatability during testing. The measurements correlated well with established functional scores.
An et al.^73^	Wearable Sensor (APDM Mobility Lab)	One hundred with DMD and 100 typically developing (2–13 years)	Left forearm, right forearm, left shank, right shank, and core region	ACC	In a clinical setting for a 14‐metre walk	The relative coupling coefficient showed worsening coordination in patients with DMD as they aged, matching clinical observations. This biomarker effectively distinguished DMD from typically developing children, suggesting its potential for early DMD screening.
Ricotti et al.^74^	Inertial measurement unit sensors (Xsens Technologies)	Twenty‐one with DMD (6–17 years) and 17 typically developing (4–16 years)	body‐suit sensors (head, shoulders, upper arms, forearms, hands, sternum, pelvis, thighs, shanks, and feet)	ACC, GYR	In a clinical setting for three visits over 12 months with assessments of activities of daily living	The use of wearable sensors was predictive of clinical scales, suggesting that sensor‐based measurements could serve as primary endpoints in DMD clinical trials and that measurements could be used to predict disease progression and track therapy response.
Rabbia et al.^75^	ActiMyo (Sysnav)	Fifty‐two with DMD (5–14 years)	Ankles	ACC, GYR, MAG	At home for 6‐week periods every 3 months, and during the clinic visits over a total of 48 weeks	The SPITFIRE/WN40227 trial was discontinued owing to lack of clinical benefit. SV95C, used as a secondary endpoint, demonstrated moderate responsiveness at week 12, while other COAs showed responsiveness at weeks 36 (6MWT) and 48 (timed 4‐stair climb, North Star Ambulatory Assessment). Strong baseline correlations and moderate to strong correlations in baseline‐to‐week 48 changes were observed between SV95C and the COAs.

Abbreviations: 6MWT, 6‐metre walk test; ACC, accelerometer; COAs, clinical outcome assessments; DMD, Duchenne muscular dystrophy; GYR, gyroscope; MAG, magnetometer; SV95C, stride velocity 95th centile.

**TABLE 8:** Summary of uses of wearable sensors in SMA, Friedreich ataxia, ataxia‐telangiectasia, Gaucher disease, LBSL, sleep disorders, and headache and migraine research.

Reference	Sensor	Population (age range)	Position	Data acquisition	Setting	Findings
*SMA*						
Montes et al.^76^	Inertial measurement unit sensors (Yost Labs)	Nine with SMA (11–51 years)	Foot	ACC	In a clinical setting during standardized gait assessments	Individuals with SMA exhibited a walking pattern characterized by a predominant use of the forefoot and a reduced initial heel strike, with the foot‐flat phase occurring early in the gait cycle. The most notable fatigue‐related change in speed was identified as a decrease in stride length.
Annoussamy et al.^77^	ActiMyo (Sysnav)	Eighty‐one with type 2 and type 3 SMA (2–30 years)	Wrist and wheelchair or wrist and ankle if patient was ambulant	ACC, GYR, MAG	At home for 2 years	Over the course of 6 months and 12 months, there was a notable decline in wrist angular velocity, wrist acceleration, wrist vertical acceleration, and power among patients diagnosed with type 2 SMA and those classified as non‐ambulant with type 3 SMA.
McIntyre et al.^78^	Opal (APDM)	One with type 1 SMA (two copies of *SMN2*) treated with nusinersen at 6 months 15 days	Ankles	ACC, GYR	At home for eight full‐day recordings across 12.5 months	Compared with typically developing infants, patients' movement throughout the day in the natural environment was significantly lower. This finding suggests that wearable sensors can effectively detect and quantify movement deficiencies in everyday life. Also, patients' movement seemed to improve with age, highlighting the promise of these sensors as objective and sensitive tools to track motor development over time.
*Friedreich ataxia*						
Mueller et al.^79^	Physilog 5 (GaitUp)	Thirteen with Friedreich ataxia and 12 typically developing (6–15 years)	Each foot, wrist, and trunk	ACC, GYR	At home for 6 days	Typically developing individuals exhibited higher activity levels derived from gait parameters than those with Friedreich ataxia.
*Ataxia–telangiectasia*						
Khan et al.^80^	GENEActiv Original actigraphy device (ActivInsights)	Fifteen with ataxia–telangiectasia and 15 typically developing (6–18 years)	Wrist	ACC	At home for 7 days	The group with ataxia‐telangiectasia displayed reduced activity levels and fewer high‐intensity movements than typically developing group. Actigraphy measures were strongly correlated with clinical severity of the disease.
Gupta et al.^81^	GENEActiv Original actigraphy device (ActivInsights)	Thirty‐one with ataxia–telangiectasia and 27 typically developing (2–20 years)	Wrist	ACC	At home for 7 days	In the group with telangiectasia, submovements were smaller, slower, and less variable in distances and speeds than in the typically developing group, suggesting an effort to attain smoother and more controlled movements.
*Gaucher disease*						
Donald et al.^82^	Million Pedometer (Aparito)	Five with Gaucher disease 1 and 16 with neuropathic Gaucher disease (13–42 years and 5–48 years respectively)	Wrist	ACC	At home for a minimum of 2 weeks and a maximum of 12 months	Wearable sensors revealed higher intensity of activity in patients with Gaucher disease 1 compared with those having neuropathic Gaucher disease.
*LBSL*						
Smith Fine et al.^83^	Opal (APDM)	Eight with LBSL (5–23 years)	Waist and on each foot	ACC	At home during remotely walking and balance tests	Patients exhibited increased gait variability and an elevated step during mid‐swing in the walking test compared with typically developing individuals.
*Sleep disorders*						
Bertoni et al.^84^	Actiwatch‐2 (Philips Respironics) and Nonin 8000 J FlexiWrap sensor (Nonin Medical)	One hundred and ninety patients (2–17 years)	Wrist and index finger respectively	ACC and oximetry respectively	In a clinical setting during full‐night polysomnography	Two wearable sensors displayed high predictive accuracy for Apnea‐Hypopnea Index >10, identifying high‐risk children who may need overnight observation.
*Headache and migraine*						
Stubberud et al.^85^	NeckSensor (EXPAIN AS), PS‐2131 (Pasco), and MIO Fuse (Mio Global, Physical Enterprises)	Ten migraine sufferers (13–17 years)	Neck, index finger, and left wrist respectively	EMG, TEMP, and ECG respectively	Usability testing in a clinical setting and at home for 2 weeks.	Data on the three physiological triggers of migraine were used to suggest self‐managed treatment through the mHealth biofeedback app. The app is safe and easy to use.
Connelly et al.^86^	Embrace (Empatica)	Thirty migraine sufferers with or without aura (10–17 years)	Wrist	ACC, EDA	At home for 4 weeks	The wearable sensor provided representative data on physical activity and autonomic arousal and was acceptable to most young patients. Participants found the wearable sensor easy to use and expressed willingness to continue using it if it could help manage their headaches. Almost all participants received notifications about possible migraine occurrences.

Abbreviations: ACC, accelerometer; ECG, electrocardiogram; EDA, electrodermal activity; EMG, electromyography; GYR, gyroscope; LBSL, leukoencephalopathy with brainstem and spinal cord involvement and lactate elevation; MAG, magnetometer; SMA, spinal muscular atrophy; TEMP, temperature.

### Cerebral palsy

Researchers have explored the use of accelerometers, pressure sensors, and inertial sensors for monitoring individuals with [CP]. In controlled environments, patients wore sensors during clinical outcome assessments. Only 3 out of 17 studies reviewed included home‐based monitoring.^6^, ^7^, ^8^ Key findings included indicators of motor capacity across different motor tasks,^9^ classification of activities of daily living,^10^, ^11^, ^12^ differentiation between CP and typically developing children,^10^, ^11^, ^12^, ^13^ and evidence suggesting that quantitative measure could predict clinical outcomes.^6^

The use of accelerometers was found to be feasible, reliable, and acceptable for assessing upper limb activity^7^ and gait parameters^14^ in patients with CP. Gross upper limb performance can be inferred from activity counts, although wrist‐worn accelerometers may not adequately capture fine finger movements.^15^ Furthermore, differences in limb usage were observed between children with unilateral CP and typically developing children; however, low‐speed rotations mistakenly increased activity counts.^13^ Owing to the ability of inertial sensors to provide less biased assessments of activity levels, they were proposed as alternatives to accelerometers.^13^

Inertial sensors tracked and quantified arm movement asymmetry across various gait speeds in patients with CP.^16^ In those with dystonic CP, sensor‐derived outcomes reliably captured pathological movements^17^ and enabled home‐based monitoring of dystonia progression.^8^ In a multi‐centre, single‐blind, randomized controlled trial, improvements in upper limb dexterity, activities of daily living, and forearm supination were observed in the intervention group following repetitive motor training.^18^ Foot‐worn inertial sensors were effective for spatiotemporal gait analysis during 200‐metre walks at self‐selected paces.^19^ Additionally, inertial sensors enhanced the accuracy and reliability of a spasticity assessment in children's lower limbs^20^ and tracked gait improvements when combined with feedback training programmes.^21^ Regarding sensor configurations for gait assessment, it was found that foot sensors are accurate for typical gait patterns in typically developing individuals, while sensors placed on the shank and thigh were more effective for moderate to severely impaired CP gait patterns.^22^ Nevertheless, detecting gait events in abnormal patterns remains challenging, particularly from the feet.^22^

### Epilepsy and seizure

Early and accurate seizure detection can improve the quality of life for patients with epilepsy by enabling better seizure management,^23^ facilitating preventive measures,^24^ reducing anxiety, and allowing greater participation in daily life activities.^25^ The consulted literature explores diverse sensor modalities, including accelerometery,^26^, ^27^, ^28^, ^29^, ^30^, ^31^, ^32^ electrocardiography,^24^, ^27^, ^28^ electrodermal activity (EDA),^23^, ^29^, ^30^, ^31^ photoplethysmography (PPG),^25^, ^26^, ^29^, ^30^, ^31^, ^33^ electroencephalography,^28^, ^34^ and electromyography^28^ to detect seizure‐related changes.

Some studies focused on detecting preictal periods. Subtle alterations in EDA signal entropy in a group of individuals with epilepsy suggest that it could serve as an indicator of seizure risk.^23^ Significant changes in PPG features during the pre‐seizure period of focal impaired awareness seizures were associated with autonomic changes related to seizures.^25^ Similarly, PPG signals during generalized tonic–clonic seizures exhibited noticeable alterations in amplitude and frequency before and after seizures.^33^

Combining accelerometery and PPG data achieved superior results compared with using only a single data type, demonstrating better‐than‐chance seizure detection for nine seizure types.^30^ A deep‐learning‐based model using accelerometer and PPG fusion reached high sensitivity, detecting 19 of 28 seizure types.^31^ Seizure forecasting, using EDA, accelerometery, body temperature, and PPG, was more accurate than random guessing in about half of the assessed patients.^29^ Performance improved with more training data, suggesting that integrating data from a wider range of patients could enhance the reliability of seizure forecasting systems.^29^

Wearable sensors have shown promise not only for general seizure detection but also for detecting typical absence seizures, reducing the review time of a 24‐hour EEG recording from 1 to 2 hours to approximately 5 to 10 minutes.^34^ In Dravet syndrome, inertial sensors were used to gain a deeper understanding of motor control impairments, with gait parameters reflecting the unsteady, ataxic gait of individuals with the syndrome.^32^

While wearable sensors have shown promise for seizure management, they do have limitations. PPG‐based systems were less reliable in detecting light reflections in individuals with darker skin tones.^26^ Additionally, adhesive patches for electromyography and electrocardiography sensors often resulted in signal loss, because their design was not suitable for paediatric patients,^28^ and wristbands sensors were sometimes removed by patients or had limited battery life.^30^

### Neurodevelopmental disorders

#### Autism spectrum disorder

When monitoring real‐world motor behaviour over prolonged periods in children aged 3 to 10 years, both with and without autism spectrum disorder (ASD), sensor variables varied by age but not by sex. Older children showed less varied movements, indicating more intentional actions. Children with ASD exhibited fewer complex movements than typically developing peers, which may help in screening for motor difficulties.^35^ Similarly, the use of inertial sensors in high‐risk populations for ASD revealed that infants who later developed ASD exhibited lower motion complexity scores.^36^, ^37^ The movement curvature, an accelerometer‐derived measure, was significantly lower in infants who were later diagnosed with ASD compared with those with attention‐deficit/hyperactivity disorder (ADHD) concerns or in the typically developing group. High movement curvature suggests more complex and variable patterns, characterized by rapid shifts between locally large and small accelerations, whereas low movement curvature indicates more gradual shifts and a less variable pattern. Lower movement variability has been shown to predict an ASD diagnosis as early as 18 months of age.^37^

In diagnosed children, repetitive behaviours such as rocking, hand flapping, and drumming were detected with high sensitivity, as were self‐injurious behaviours, which could help in treatment selection and monitoring.^38^, ^39^, ^40^ Moreover, daily gestures typically performed by children with ASD were classified with the aid of machine learning algorithms, aiming to enhance the understanding of their needs and emotions.^41^

Treating anxiety in individuals with ASD remains a challenge due to difficulties with self‐awareness and communication of anxiety symptoms.^42^ Wearable sensors were used to monitor physiological arousal in real‐time, demonstrating feasibility for anxiety detection and management.^42^ Additionally, wearable sensors were used to collect multimodal signals that could predict precursors to problematic behaviours, with body motion being the most predictive sensing modality.^43^

Classroom performance can be improved by predicting stress and concentration levels using heart rate and accelerometer data.^44^ Heart rate signals can also categorize positive, negative, or neutral emotional states during avatar interactions, although frequent hand movements compromised signal quality.^45^

#### ADHD

Combining motion sensor output with patient‐reported activity levels achieved high diagnostic accuracy in distinguishing typically developing children from those with ADHD through the application of machine learning.^46^ Another study found that children with ADHD presented daytime hyperactivity, but no substantial sleep disruptions compared with typically developing peers.^47^

Actigraphy, in conjunction with recurrent neural networks, demonstrated that medication influence movement intensity in ADHD.^48^ Compared with children who did not have ADHD, unmedicated individuals with ADHD exhibited significant distinct movement patterns at medium intensities, while those on medication showed statistically different behaviours at lower intensities.^48^ Wearable‐sensor‐derived physical activity data and machine learning have also been highly effective in differentiating between physically aggressive and non‐aggressive behaviour, indicating potential for remote monitoring and management.^49^

Despite these promising findings, challenges related to battery life^46^, ^49^ and interface were observed.^46^ Compliance issues included discomfort with wearing the device,^47^, ^49^ reluctance to wear the device at school, and occasional malfunctions.^47^

#### Rett syndrome

Wearable sensors have shown feasibility in continuously monitoring heart rate, respiratory rate, EDA, and movement in Rett syndrome (RTT), despite challenges such as wearability issues, operator errors, and data noise.^50^, ^51^, ^52^ In RTT, heart rate is higher during the day and lower at night across all ages.^53^ Those with RTT also have less heart rate variability (HRV), a higher low‐frequency to high‐frequency ratio, and disrupted circadian regulation compared with typically developing individuals, which are signs of autonomic dysfunction.^53^, ^54^ Age‐related HRV metrics show higher values in paediatric patients compared with adolescents and adults.^50^, ^53^ Although a recent study did not support previous findings of reduced HRV in the population with RTT, it identified two variables, the percentage of maximum heart rate and the heart rate to HRV low‐frequency power, as potential objective markers of fatigue.^50^ Both metrics were elevated, independent of the wake–sleep cycle, indicating persistent fatigue in patients with RTT.^50^ HRV, respiration rate, and skin temperature may also be sensitive to *MECP2* gene mutations, particularly in patients with early truncation variants.^50^, ^54^ Significant differences in respiratory rate and skin temperature were observed across clinical stages of RTT as disease progressed.^50^

EDA normalization was linked to symptom improvement in four patients with RTT treated with buspirone, suggesting its potential as a biomarker for dysautonomia.^51^ Additionally, machine learning models using sensor data predicted ASD/Rett status with 95% accuracy and classified sleep stages in RTT with 85.1% accuracy using accelerometers, EDA, and PPG.^52^, ^55^

#### Down syndrome, Angelman syndrome, and Prader–Willi syndrome

Despite evidence suggesting different walking and postural strategies between adults with Down syndrome and Prader–Willi syndrome, children often receive similar treatments. Spatiotemporal gait parameters can differentiate children with Down syndrome, Prader–Willi syndrome, and typical development, which may help clinicians create a more individualized clinical picture.^56^ Sensors may also be valuable for assessing the effectiveness of early interventions in Down syndrome, as demonstrated by an increase in leg movements following kicking sessions in infants under 5 months of age.^57^

Ankle‐worn sensors enable accurate real‐world gait evaluations for individuals with Angelman syndrome and Prader–Willi syndrome that closely match laboratory tests.^58^ The use of wearables in both ankles was well tolerated in another population with Angelman syndrome, with participants recording several hours of data per day.^59^ Gait analysis indicated reduced walking distances, shorter strides, longer median stride times, lower stride velocity 95th centile (SV95C), and less variable speeds than typically developing comparison individuals.^59^

### Neuromuscular disorders

#### Duchenne muscular dystrophy

Several therapeutics are currently being evaluated in clinical trials for the treatment of DMD.^87^ However, traditional assessment methods often struggle to reliably capture significant changes in a patient's condition over the typical 1‐year trial period.

Accelerometers can track various physical activity parameters such as steps, distance, step frequency, gait speed, activity levels,^60^, ^61^, ^62^, ^63^, ^64^, ^65^, ^66^, ^67^, ^68^, ^69^ and upper limb movement.^68^, ^69^, ^70^, ^71^, ^72^ Sensors placed on different body parts (chest, arms, wrists, hips, and ankles) provide valuable insights into daily movement patterns, aiding in the assessment of disease progression and therapeutic response.^60^, ^63^, ^66^, ^67^, ^68^, ^69^, ^70^, ^88^

Studies comparing gait in patients with DMD and typically developing comparison individuals revealed that those with DMD exhibit fewer daily steps, slower step velocity, reduced trunk movement, and lower coordination, suggesting that wearable sensors might also be useful for DMD screening.^61^, ^62^, ^63^, ^64^, ^68^, ^73^, ^74^

The SV95C was the first digital endpoint approved by a regulatory agency, initially as a secondary^89^ and later as a primary endpoint^90^, ^91^ for DMD clinical trials. The qualification of the SV95C was facilitated by the feasibility of using magneto‐inertial sensors on both ankles in children aged 4 years and older for long periods in uncontrolled environments. The SV95C captures the velocity of the 5% most rapid strides performed by an individual, and its approval by the European Medicines Agency was based on demonstrated reliability, responsiveness to change, and correlation to well‐established clinical outcome measures in DMD. SV95C values in typically developing children have shown no dependence on age or height, allowing growth to be excluded as a variable in year‐long clinical trials.^91^ A practical example of its sensitivity to change in clinical trials is the SPITFIRE/WN40227 trial, where SV95C detected functional decline earlier than traditional assessments.^75^

Additionally, integrating artificial intelligence with the collected data enables the prediction of clinical scales, gait parameters, patient classification, and disease progression.^61^, ^62^, ^74^

#### SMA

The introduction of disease‐modifying therapies for SMA has transformed the care of patients, improving symptoms in those diagnosed after symptom onset and being transformative for those identified through newborn infant screening.^92^ Given the high cost of these treatments^93^ and the rise of combination therapies,^94^ precise measurement of patient improvement is essential.

Wearable sensors facilitate the measurement of stride length, walking velocity, and endurance, offering valuable insights into muscle fatigue and walking patterns in patients with SMA.^76^ Furthermore, continuous monitoring of upper limb movement in patients with SMA types II and III using magneto‐inertial sensor metrics can detect disease progression earlier than traditional methods.^77^ These sensors are also useful in measuring variations in leg movement patterns over time and distinguishing between children with SMA and typically developing individuals, as movement rates are lower in patients with SMA.^78^

### Ataxia

#### Friedreich ataxia

A single study has investigated the use of wearable sensors to assess gait, balance, and arm movements in individuals with Friedreich ataxia within a home environment. Participants exhibited significant lower activity levels than typically developing comparison individuals, particularly in foot and wrist movements. Cadence was strongly correlated with the Timed 25‐foot walk test?, while stride width was significantly associated with fall risk. Peak swing and stance periods provided the most discriminatory insights between patients with Friedreich ataxia and typically developing comparison individuals?. However, upper limb data were more challenging to interpret owing to the complexities and variability of everyday actions, making classification into specific activities of daily living difficult. Additionally, mean stride width showed a significant correlation with GAA repeat length of the short allele, while no significant relationship was found between allele length and Timed 25‐foot walk time in the same participants.^79^

#### Ataxia–telangiectasia

In ataxia–telangiectasia, wrist‐worn accelerometers have been used to assess motor impairment and disease progression.^80^, ^81^ Findings indicate that individuals with ataxia–telangiectasia exhibit lower activity levels and fewer high‐intensity movements than typically developing comparison individuals, with activity metrics correlating with the clinical severity.^80^ Additionally, patients with ataxia–telangiectasia demonstrated smaller, slower, and less variable submovements—segmented wrist movements during larger voluntary actions—than typically developing peers, suggesting an effort to achieve smoother and more controlled movements. Over the course of 1 year, no significant changes were observed in the sensor data of the comparison group, whereas in the ataxia–telangiectasia group, sensor data changes aligned with disease progression, as confirmed by the Balance Assessment Rating Scale.^81^

### Neurometabolic diseases

The study of walking patterns using a wrist‐worn accelerometer indicated that individuals with neuronopathic Gaucher disease had lower activity levels than patients with type 1 Gaucher disease. This difference may be attributed to symptoms such as ataxia and tremors, which interfere with the ability to perform intense activities that require both strength and coordination.^82^

Furthermore, the analysis of gait and balance in patients with leukoencephalopathy involving the brainstem and spinal cord and lactate elevation revealed that gait variability and step elevation at mid‐swing were higher compared with age‐matched typically developing individuals during brief walking tests.^83^

### Sleep disorders

The application of machine learning to actigraphy and oximetry has improved the accuracy in identifying children with severe obstructive sleep apnoea syndrome compared with predictions made using only clinical parameters, suggesting that wearable sensors could be used for home‐based screening for overnight observation.^84^

### Headache and migraine

Sensors can continuously monitor not only physiological data but also surrounding environmental data, providing valuable insights into headache mechanisms. One study focused on using multi‐sensor data to provide feedback during migraine prophylaxis, while a second study integrated physiological data with environmental information to identify factors influencing headache episodes.^85^, ^86^ Although the methods for data acquisition seem to be acceptable for patients, further work is needed to refine the results. Reasons for incomplete wearable sensor data included technical issues in data acquisition and participant‐related factors, such as sensors not recording data even though participants claimed to have worn the band and taking off the device for charging or forgetting to wear it.^86^

## DISCUSSION

Wearable sensors hold promise for transforming paediatric neurology by providing a non‐invasive, long‐term method for monitoring disease progression and offering guidance to improve treatment. These devices can collect a wide range of physiological and activity‐related data, allowing for the earlier detection of subtle changes that may be missed by standard clinic assessments, ultimately resulting in improved outcomes through timely interventions.^30^, ^59^ Moreover, home‐based monitoring may increase patients' participation in trials and care accessibility, reducing the need for frequent clinic visits and relieving patients from stressful assessments.^95^ Wearable sensors also offer an advantage in assessing very young children or infants with intellectual disabilities, with whom active and reliable collaboration may be challenging.

The impact of wearables devices is evident in DMD, where the SV95C metric became the first digital outcome measure to received regulatory approval across all fields of medicine.^91^ The sensitivity to change of the SV95C highlights how wearables can improve future clinical trial design, enabling shorter and less expensive trials, without compromising the power of the study. Earlier drug launches not only benefit patients but also result in higher asset lifecycle values for pharmaceutical companies.^96^ Further evidence of regulatory interest in wearable technologies is the Apple Watch's Atrial Fibrillation History feature, which became the first digital tool qualified under the US Food and Drug Administration's Medical Device Development Tools program in May 2024 and is now recognized as a secondary endpoint in studies of cardiac ablation devices.^97^ As this trend persists, projections suggest widespread adoption of wearables in clinical trials, with up to 70% of trials expected to incorporate wearable sensors in 2025.^95^

Artificial intelligence can analyse complex data sets, identify patterns, and make predictions. For instance, in epilepsy management, artificial intelligence has been used to predict seizure occurrences and provide timely warnings, improving patients' quality of life.^24^ However, the application of artificial intelligence is often limited by the need for large data sets,^29^, ^44^, ^46^, ^48^, ^62^ which can be difficult to obtain, particularly for rare neurological conditions. Furthermore, evaluating the clinical relevance of outcomes derived from artificial intelligence is extremely difficult owing to the lack of standardization in evaluation data sets, task definitions, and performance metrics. Therefore, it is essential to develop validation frameworks that build on existing standards and guidelines.^98^

The studies reviewed reveal several challenges that may affect the use of wearable sensors. A primary concern is the population size, as many were unable to validate their results owing to a limited number of participants. The difficulty in recruitment is further aggravated by disease progression, leading to patients' withdrawal due to factors such as mortality, loss of ambulation, or participation in other treatment trials.^60^, ^77^ Non‐adherence and compliance issues may also arise, as patients may forget to wear the devices or find them uncomfortable, inconvenient, or aesthetically unappealing, leading to discontinuation.^60^ Additionally, technical and environmental challenges with the sensors themselves can exacerbate compliance issues.^71^, ^80^, ^86^, ^95^ Operator‐related errors, such as incorrect positioning or loose fitting of the wristbands, may affect the quality of signals, requiring extensive training for patients.^51^, ^52^ The regulatory approval process for digital outcomes can be lengthy and demand substantial data about patients to establish objectivity, reliability, and sensitivity to change.^88^, ^99^ In some cases, corporate investment may not be viable given the limited size of the potential market. Furthermore, many studies have used wearables for short, 1‐day recordings, which may be biased by patients' motivation, mirroring challenges faced with in‐clinic assessments. For instance, a child diagnosed with SMA exhibited atypical increase in movement rates during a single recording session, probably because of the excitement of being at a park.^78^ This example emphasizes the importance of long‐term monitoring strategies to capture more accurate and nuanced data about disease progression.

The increasing use of wearable sensors in paediatrics raises ethical concerns about invasiveness of privacy, access, and surveillance. In long‐term monitoring studies, pseudo‐anonymization may be insufficient, as re‐identification is possible.^100^, ^101^ Continuous monitoring of sensitive data, such as location, necessitates strict limitations on data processing to safeguard privacy while balancing technological benefits, such as alerts for potential health crises including location information.^100^ Moreover, wearable devices can inadvertently collect data from non‐users through cameras or microphones, further complicating privacy issues.^100^ Concerns also arise about who accesses the data and its intended use, with healthcare providers expected to watch over patients' well‐being while technology companies may prioritize commercial interests.^100^ To protect patients' privacy and prevent misuse of sensitive information, transparency, confidentiality, and clear limitations on data use are needed.^100^, ^101^ Disparities in technology access because of socioeconomic factors also pose risks, as they can lead to underrepresentation of certain groups, resulting in biased outcomes.^100^ This can result in algorithms that underperform or overperform for specific demographics, as seen in the poorer performance of PPG in measuring blood flow in individuals with darker skin tones.^26^ While wearable sensors can provide valuable health insights and enhance psychological well‐being, the risks associated with inaccurate data must be mitigated, as they can have physical, emotional, and psychological consequences.^100^ To address these concerns, clinical studies should assess the feasibility, sensitivity, and specificity of these devices. However, in paediatric trials, informed consent may be questionable, as children may not fully comprehend the implications of data collection. Additionally, wearable sensors can impose burdens on participants, including physical discomfort, psychological stress, stigma, or interference with daily activities.^101^

Further research is necessary to explore how these technologies can be effectively integrated into clinical practice, in ways that minimize interference, optimize benefits for patients, and reduce the long‐term risks associated with the passive collection of sensitive, emotional, behavioural, and neural data.^101^ Alongside ethical concerns, the clinical implementation of wearable sensors in paediatric neurology remains limited because of continuing validation processes, regulatory hurdles, and difficulties in integration into standard care. Currently, the use of wearable sensors varies in maturity, with most work focused on proving feasibility and reliability. Longitudinal studies and user‐centred designs to improve usability and comfort for paediatric patients, along with collaboration between clinicians, engineers, and regulators, are crucial to expanding acceptance, development, and clinical utility.^102^

This review has limitations. First, papers were selected by a sole individual, and our search was restricted to a single database. This approach may have introduced biases and overlooked pertinent studies accessible through alternative databases or selection methods. Additionally, a total of eight papers were not accessible. Nonetheless, our review demonstrates that wearable technologies are not restricted to adult neurological conditions such as multiple sclerosis, Parkinson disease, or amyotrophic lateral sclerosis but can be essential components of assessment of paediatric patients with neurological conditions.

## CONCLUSIONS

More research is needed to prove the effectiveness of interventions based on wearable sensors and their impact on long‐term outcomes for patients. Collaboration among researchers, clinicians, and technology developers will be essential in overcoming challenges and maximizing the full potential of wearable sensor technology in paediatric health care. However, recent advancements such as the regulatory validation of the SV95C metric and its adoption as a primary endpoint in clinical trials demonstrate considerable progress. We expect wearable sensors to revolutionize the diagnosis, monitoring, and management of neurological conditions in children.

## CONFLICT OF INTEREST STATEMENT

CGB is an employee of the ActiMyo/Syde wearable sensor manufacturer, Sysnav. LS gave consultancy to Roche, Biogen Digital Health, PepGen, Dyne Therapeurics, WaveLife and Sysnav in the context of digital outcome measures.

## FUNDING INFORMATION

The authors received no specific funding for this work.

## Data Availability

Data sharing not applicable ‐ no new data generated.
